# Elucidating Sensorimotor Control Principles with Myoelectric Musculoskeletal Models

**DOI:** 10.3389/fnhum.2017.00531

**Published:** 2017-11-10

**Authors:** Sarah E. Goodman, Christopher J. Hasson

**Affiliations:** ^1^Neuromotor Systems Laboratory, Department of Bioengineering, Northeastern University, Boston, MA, United States; ^2^Neuromotor Systems Laboratory, Department of Physical Therapy, Movement and Rehabilitation Sciences, Northeastern University, Boston, MA, United States; ^3^Neuromotor Systems Laboratory, Department of Biology, Northeastern University, Boston, MA, United States

**Keywords:** motor learning, motor control, musculoskeletal, neuromusculoskeletal, modeling, sensorimotor control, biomechanics

## Abstract

There is an old saying that you must walk a mile in someone's shoes to truly understand them. This mini-review will synthesize and discuss recent research that attempts to make humans “walk a mile” in an artificial musculoskeletal system to gain insight into the principles governing human movement control. In this approach, electromyography (EMG) is used to sample human motor commands; these commands serve as inputs to mathematical models of muscular dynamics, which in turn act on a model of skeletal dynamics to produce a simulated motor action in real-time (i.e., the model's state is updated fast enough produce smooth motion without noticeable transitions; Manal et al., 2002). In this mini-review, these are termed myoelectric musculoskeletal models (MMMs). After a brief overview of typical MMM design and operation principles, the review will highlight how MMMs have been used for understanding human sensorimotor control and learning by evoking apparent alterations in a user's biomechanics, neural control, and sensory feedback experiences.

## Myoelectric musculoskeletal model design and operation

### Basic components

As the name implies, an essential component of an MMM is a musculoskeletal model: a mathematical representation of bones, muscles, and connective tissue. The model specifics depend on the application (Winters, 1995). For simulation of gross motor activities, many MMMs use Hill-type lumped parameter muscle models (Hill, 1938) and rigid-body skeletal models due to their computational speed, though this is becoming less of an issue with advances in computing power. More dynamically complex models may be needed for some applications, e.g., when considering complex muscle fiber architecture (Blemker and Delp, 2005; Heidlauf and Röhrle, 2014), stress and stress and strain distributions within bone (Huiskes and Chao, 1983), or the heterogeneity of fiber architecture within a muscle (Röhrle et al., 2017). As several comprehensive reviews are available on the possibilities for modeling the musculotendon system, the reader is pointed to the literature for more information (Zajac, 1988; Zajac and Winters, 1990; Neptune, 2000; Pandy, 2001; Viceconti et al., 2006).

### Controlling the model

For a musculoskeletal model to produce functional movement the muscle models must be activated in a coordinated fashion; this is typically achieved in one of two ways. The first is by using a central nervous system (CNS) model that codifies the principles governing human movement control. In such models, muscle activation patterns are generated by minimizing a quantity of interest such as energy (Hatze and Buys, 1977), muscle stress (Crowninshield and Brand, 1981a), or movement smoothness (Hogan, 1984). These models and derivatives thereof have been useful in gaining insight into the high-level operations of the CNS for movement control and production (Todorov, 2004). The second way to control a musculoskeletal model is by using measured muscle activity (i.e., EMG). This can occur off-line, for example when MMMs are used to estimate muscle force distributions. Alternatively, a musculoskeletal model can be controlled on-line, such that a user interacts with the MMM in real-time (see section Using Myoelectric Musculoskeletal Models to Elucidate Motor Control Principles for examples of off- and on-line approaches). The on-line approach is best suited for use in motor control and learning experiments, and is therefore the focus of this mini-review.

### Personalizing the model

How a human controls a MMM depends on its programmed dynamics, such as muscle mechanical properties and moment arm-joint angle relations. Early modeling approaches used musculoskeletal models with cadaver- and animal-based biomechanical properties (e.g., Anderson and Pandy, 2001); however, the behavior of a musculoskeletal model is sensitive to many biomechanical properties (Scovil and Ronsky, 2006; Redl et al., 2007; De Groote et al., 2010; Ackland et al., 2012; Pau et al., 2012). Therefore, techniques have been developed to personalize model components, e.g., by using a dynamometer to quantify maximum muscular torque capability (Garner and Pandy, 2003), MRI to measure muscle volumes to estimate maximal isometric force (e.g., Maganaris et al., 2001; Hasson and Caldwell, 2012), or ultrasound to measure musculotendon series-elastic stiffness (e.g., Kubo et al., 1999; Hasson et al., 2011). While the importance of model personalization ultimately depends on the application, it may be particularly important for increasing the realism of MMMs (further discussed in section Current Challenges and Future Directions).

## Using myoelectric musculoskeletal models to elucidate motor control principles

**Figure 1:**
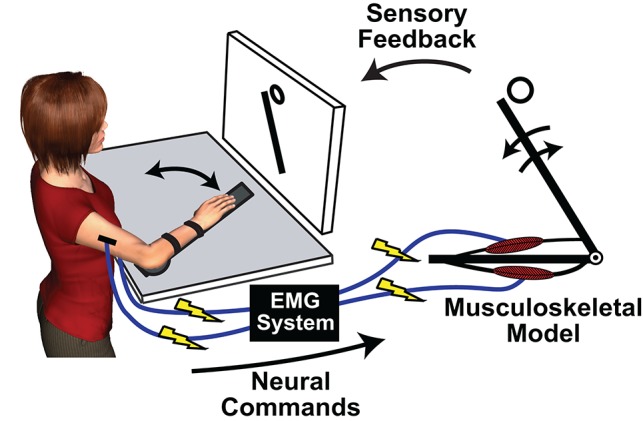
An exemplary implementation of a myoelectric musculoskeletal model (MMM) for investigating sensorimotor control principles. © [2017] IEEE. Adapted/reprinted, with permission, from Hasson (2017).

One of the first uses of MMMs was to solve the problem of resolving a measured net joint torque into individual muscular force components (Crowninshield and Brand, 1981b). This can be accomplished by driving a musculoskeletal model with measured EMG, i.e., performing an off-line forward simulation with a MMM, and using optimization techniques to determine the set of individual muscle forces that sum to match the net joint torques (Manal et al., 2002; Lloyd and Besier, 2003; Chadwick et al., 2009; Pau et al., 2012; Sartori et al., 2012). More recently, researchers have started to exploit a unique property of MMMs: they allow a virtual decoupling between the human CNS and the controlled biomechanics. Through an MMM an individual can be presented with novel neuromusculoskeletal dynamics, and adaptations to these dynamics can inform hypotheses regarding motor learning and control. Typically in the MMM paradigm, surface-mounted EMG electrodes record control signals to drive a musculoskeletal model while the actions of the model are displayed on a monitor (Figure 1). It is also possible to physically impose the actions of the model back onto the user with a robotic device (Hasson, 2017). The following sections review recent studies using MMMs to investigate human adaptations to manipulations of muscle coordination strategies, neural dynamics, and sensory feedback.

### Adaptation to altered muscular activation vs. force mappings

A major use of MMMs has been to examine how the CNS solves the problem of motor redundancy, i.e., how to activate muscles to perform an action, given that there is usually an infinite number of muscular force time history combinations that produce the action (Bernstein, 1967). Note that this can be viewed as a “feature” instead of a “bug,” providing the CNS with the flexibility to deal with constraints imposed by a task, fatigue, or environmental disturbances (Latash, 2012). Knowing how the CNS controls its redundant musculature is of interest for rehabilitation, as a similar problem exists in the control of advanced prostheses and exoskeletons (Farina et al., 2014, 2017; Ison and Artemiadis, 2014). Many posit that the brain solves this problem by optimizing behavior relative to a performance criterion (Todorov and Jordan, 2002; Todorov, 2004; Haruno and Wolpert, 2005). Others hypothesize that muscular coordination is framed by neural constraints in the form of motor primitives or muscular synergies (Lee, 1984; Macpherson, 1991; Todorov and Jordan, 2002; d'Avella et al., 2003; Hogan and Sternad, 2012; Bizzi and Cheung, 2013; Giszter, 2015) and by habitual coordination patterns (de Rugy et al., 2012a,b).

MMMs are uniquely positioned to tackle questions related to the principles underlying human movement control because they can expose an individual to novel, yet fully specified (i.e., programmed), biomechanics. Capitalizing on this strength, de Rugy et al. (2012a) used an MMM to examine how the CNS coordinates force-sharing among synergistic muscles. They asked subjects to control a forearm MMM and produce a net MMM force in a specific direction, which depended on the biomechanical arrangement of simulated forearm muscles. Interestingly, after disabling one of the simulated muscles, subjects did not re-optimize their muscular activation patterns, leading the authors to conclude that there is a strong habitual component to neuromotor control that may override CNS optimization processes. A related study by Berger et al. (2013) used an MMM to test the hypothesis that muscular synergies reflect a set of basic control modules, instead of a simple manifestation of task or biomechanical constraints. The results showed that humans could learn a novel task faster if it required a recombination of existing synergies compared to new/non-native synergies, supporting an inherent modularity to muscular activation. Although this work focuses on motor synergies, it is worth pointing out that there may be synergies in sensory information processing as well (Latash, 2008; Alnajjar et al., 2014; Damiano, 2015); future work with MMMs could help shed light in this domain. Understanding the organization and modulation of muscle synergies may better inform neurorehabilitation, robotics, and technologies that rely on accurate and timely decoding of the kinetics and kinematics of movement plans (Ison and Artemiadis, 2014).

The studies above have provided valuable insights into how the CNS controls movement; however, the simulated muscles functioned primarily as pure force generators. In reality, muscles have various mechanical properties that influence the translation between EMG and muscular force. In contrast to limb dynamics (Sainburg et al., 1999), less is known about how the CNS represents muscular dynamics in movement control. An MMM is well-suited to perturbing muscular dynamics, as done by one study that used an MMM to test whether humans learn a novel task faster using individualized muscle models or more artificial force generators (Hasson, 2014). The expectation was if participants had a neural representation of muscle dynamics, it would offset any learning challenges associated with the more dynamically complex muscle model. This was indeed the case, as the muscle model group improved performance (movement speed and accuracy) as fast as the force generator group with improved generalization. Further research in this area is warranted because it is relevant to prosthetics, some of which include artificial muscular dynamics (e.g., Eilenberg et al., 2010).

### Adaptation to altered neural dynamics

MMMs are also useful for testing theories of how humans adapt to the properties of the nervous system itself. An important property of motor commands is that they include signal-dependent noise (SDN), i.e., the variance is proportional to the command magnitude (Clamann, 1969; Matthews, 1996). It has been theorized that the CNS is aware of this feature and optimizes its movement control to minimize the effects of SDN (Harris and Wolpert, 1998; Jones et al., 2002). This hypothesis has been supported by simulations of computational models with output closely resembling human movement data (Harris and Wolpert, 1998; Jones et al., 2002; Todorov and Jordan, 2002; van Beers et al., 2004). An alternative and arguably more direct, approach for testing this theory is to modify SDN in humans and observe if they respond in a way that is consistent with the optimal control hypothesis. While this is challenging to do experimentally, it can be done expeditiously with an MMM.

Such an approach was taken by de Rugy et al. (2012a), who increased the variability of the force output of a single simulated muscle within an MMM during an isometric forearm force-production task. According to the theory that humans behave optimally to reduce SDN effects, if one muscle's force output is made more variable the CNS should reduce activation of that muscle to minimize SDN (Harris and Wolpert, 1998; Jones et al., 2002). As for the disabling of a simulated muscle discussed earlier, the results showed that subjects did not change their behavior in response to the increased variability, which may further support the hypothesis that human sensorimotor control is strongly habitual, or it could be that subjects failed to respond to the virtual manipulation in a realistic way (see section Current Challenges and Future Directions for further discussion). A study by Hasson et al. (2016) employed an MMM to investigate human adaptation to SDN manipulations using a dynamic point-to-point arm movement; however, in this case, subjects modified their behavior by increasing antagonistic co-activation. This could be because, in contrast to de Rugy and colleagues, the simulated arm task was dynamic rather than isometric, and antagonistic co-activation can reduce kinematic variability (Selen et al., 2005; van Dieen et al., 2008; Ueyama and Miyashita, 2013). Although the discussed results are mixed, this research highlights the possibilities for using MMMs to test sensorimotor control hypotheses, and shows that subject responses are sensitive to the constraints imposed on both the real and virtual arms.

### Adaptation to sensory feedback manipulations

We will lastly touch upon the utility of MMMs is to gain insight into the effects of sensory feedback manipulations on movement control. Most studies using MMMs in this capacity focus on proprioceptive information because an MMM serves as a natural virtual prosthesis, and proprioception is typically impoverished with prosthetic devices (Antfolk et al., 2013). In this section, the definition of MMM is relaxed because few of the reviewed studies use prostheses that are both myoelectrically-driven and include explicit models of musculotendon dynamics (a fruitful avenue for future exploration). While it is possible to provide direct proprioceptive feedback to existing sensory afferents of amputees (Dhillon and Horch, 2005), this mini-review will focus on non-invasive approaches utilizing electrotactile, vibrotactile, and skin-stretch stimulation, as these have been used in conjunction with MMMs (Antfolk et al., 2013).

Many studies using MMMs or prosthetic arms have concentrated on kinetic feedback because interaction forces are critical for object manipulation yet cannot be directly perceived with vision (Chiel and Beer, 1997). Early studies provided grasping force feedback with electrotactile stimulation (Prior et al., 1976; Scott et al., 1980). While still under investigation (Dosen et al., 2017), the discomfort associated with electrical stimulation led to exploration of vibrotactile stimulation applications. Several studies have shown that vibrotactile force feedback improves performance with both simulated (MMMs) and real myoelectric prosthetic arms (Patterson and Katz, 1992; Pylatiuk et al., 2006; Chatterjee et al., 2008a,b; Stepp and Matsuoka, 2010; Rombokas et al., 2013). However, the degree of improvement varied, and actions often became slower with vibrotactile feedback (Stepp and Matsuoka, 2010; Witteveen et al., 2012b). From a human sensorimotor control point of view it is important to account for the speed-accuracy tradeoff (i.e., Fitt's Law), when assessing motor skill (Woodworth, 1899; Fitts, 1954), as it is easier to maintain accuracy if movement is slowed (Shmuelof et al., 2012). Nonetheless, this research has demonstrated the plasticity of the CNS and its capacity to perceive and integrate non-native sensory information through alternative sensory channels when using an MMM.

Since motion can be visually perceived, kinematic vibrotactile stimulation should be most beneficial for MMM control when vision is absent. This has indeed been shown in studies using vibrotactile stimulation to convey hand position (Witteveen et al., 2012a; Christiansen et al., 2013), and some have reported benefits even when vision remains available (Sergi et al., 2008). Others have shown that conveying goal-related error information with vibrotactile feedback is beneficial (Bark et al., 2015; Tzorakoleftherakis et al., 2016; Krueger et al., 2017). While these studies have used relatively slow movements, the advantages of kinematic vibrotactile feedback during fast MMM motions are less clear: to date no benefits have been shown (Bark et al., 2011; Hasson and Manczurowsky, 2015). This could be because rapid movements are predominantly open-loop and rely more on an internal model of musculoskeletal dynamics (Kawato, 1999). Finally, MMM kinematics can also be signaled using skin-stretch feedback, which has been shown to improve MMM control (Bark et al., 2008; Wheeler et al., 2010). These investigators hypothesize that skin stretch might be more intuitive than electro/vibrotactile feedback due to prior evidence that skin stretch information contributes to kinesthesia (Edin and Johansson, 1995).

## Current challenges and future directions

**Figure 2:**
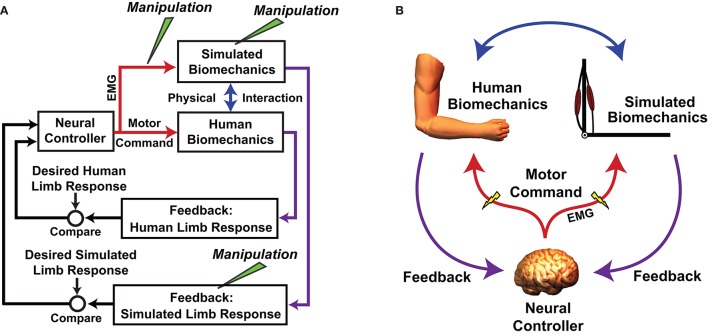
Flowchart **(A)** and schematic **(B)** showing how the real neuromuscular system is embedded how the real neuromuscular system is embedded in a myoelectric musculoskeletal model (MMM). Both systems act in parallel and are driven by the same neural controller (the human central nervous system [CNS]). Neural commands are sampled with electromyography (EMG) before being digitized and used to control a musculoskeletal model (in this example the arm is used). The CNS receives feedback about the states of both the actual and simulated biomechanics. The physical interaction can be rigid with the human limb fixed in space, or the actions of the simulated system can be imposed onto the actual system using a mechanical apparatus. Simulated manipulations can be performed at various points in the control loop. **A** is adapted/reprinted, with permission, from Hasson (2014); ©[2014] Springer-Verlag Berlin Heidelberg.

An important caveat of using MMMs to test motor adaptation hypotheses is that an MMM acts in parallel with the actual neuromusculoskeletal system (Figure 2) and simulated manipulations do not alter a user's neuromuscular substrate. This is both a strength and weakness of the approach; the manipulations do no harm, but it can be difficult to determine the degree to which an individual's adaptation to a virtual manipulation reflects reality. Nevertheless, there are avenues available for validating virtual outcomes. For example, in the study by de Rugy et al. (2012a), subjects' responses to a simulated muscle deletion were compared with those in response to exercise-induced fatigue of the same muscle (the results agreed). In reality, it may not always be possible to perform an analog manipulation such as virtually rearranging muscle origins and insertions. Future studies may explore alternative ways of improving confidence in MMM experimental outcomes, such as by increasing the realism of an MMM through steps such as personalization of model parameters and improving proprioceptive feedback (Hasson, 2017). This follows the logic that if a human controls an MMM that is indistinguishable from his/her own arm, or at least has very similar dynamics, the response to MMM manipulations should be a close reflection of reality.

Future research should also investigate the ramifications of limb motion when controlling MMMs. While some studies maintain both real and simulated limbs in an isomeric state (e.g., de Rugy et al., 2012a,b; Berger et al., 2013), others keep the real limb restrained but allow the simulated limb to move (e.g., Hasson and Manczurowsky, 2015; Hasson et al., 2016; Krueger et al., 2017). The rationale for keeping the real arm fixed is that it limits movement artifacts from contaminating EMG signals driving the MMM (De Luca et al., 2010). Recent evidence suggests that some of what is learned under isometric conditions transfers to non-isometric conditions (Melendez-Calderon et al., 2017), but as most actions in the real world involve limb movement, allowing the user's arm to move could significantly increase MMM realism. However, unless the real and simulated limb dynamics are well-matched they will move asynchronously and proprioceptive information will be incongruent. One solution is to use a motor to force the real arm to match the simulated arm motion. This was originally done for studies on proprioception (Kuchenbecker et al., 2007; Blank et al., 2010) and recently implemented in an MMM by Hasson (2017). Interestingly, Hasson showed that given a properly personalized MMM, users could reach a relatively high level of performance in a dynamic motor task. This suggests that proprioception could be a limiting factor in the control of EMG-driven prostheses, in addition to uncertainties introduced with EMG-control (Johnson et al., 2016). Nonetheless, even if the real vs. simulated limb motion is robotically matched at the joint level, there may still be proprioceptive mismatches at the muscle level, which should be explored in future research.

## Conclusions

This mini-review addressed the ways in which MMMs can serve as tools to probe fundamental questions in human sensorimotor control and learning including CNS coordination of force-sharing among muscles, adaptation to modifications of neural dynamics, and the limits of sensory feedback augmentation. The power of MMMs stems from the experimental control they offer: model dynamics are mathematically specified and are therefore known quantities, and manipulations can be performed selectively to control for confounds associated with real neuromuscular interventions. The main limitation is the challenge of validating human adaptations to simulated manipulations given that experimental analogs are often impossible. Nevertheless, the knowledge gained from creatively employing MMMs in research has significant implications for fields such as neuroscience, biomechanics, engineering, and rehabilitation.

## Author contributions

All authors listed have made a substantial, direct and intellectual contribution to the work, and approved it for publication.

### Conflict of interest statement

The authors declare that the research was conducted in the absence of any commercial or financial relationships that could be construed as a potential conflict of interest.
